# Molt-inhibiting hormone stimulates vitellogenesis at advanced ovarian developmental stages in the female blue crab, *Callinectes sapidus *2: novel specific binding sites in hepatopancreas and cAMP as a second messenger

**DOI:** 10.1186/1746-1448-5-6

**Published:** 2009-07-07

**Authors:** Nilli Zmora, Amir Sagi, Yonathan Zohar, J Sook Chung

**Affiliations:** 1 Center of Marine Biotechnology, University of Maryland Biotechnology Institute, 701 E Pratt St Columbus Center, Suite 236, Baltimore, MD 21202, USA; 2 Department of Life Sciences and the National Institute for Biotechnology in the Negev, Ben Gurion University, Beer Sheva, Israel

## Abstract

The finding that molt-inhibiting hormone (MIH) regulates vitellogenesis in the hepatopancreas of mature *Callinectes sapidus *females, raised the need for the characterization of its mode of action. Using classical radioligand binding assays, we located specific, saturable, and non-cooperative binding sites for MIH in the Y-organs of juveniles (J-YO) and in the hepatopancreas of vitellogenic adult females. MIH binding to the hepatopancreas membranes had an affinity 77 times lower than that of juvenile YO membranes (K_D _values: 3.22 × 10^-8 ^and 4.19 × 10^-10 ^M/mg protein, respectively). The number of maximum binding sites (B_MAX_) was approximately two times higher in the hepatopancreas than in the YO (B_MAX _values: 9.24 × 10^-9 ^and 4.8 × 10^-9 ^M/mg protein, respectively). Furthermore, MIH binding site number in the hepatopancreas was dependent on ovarian stage and was twice as high at stage 3 than at stages 2 and 1. SDS-PAGE separation of [^125^I] MIH or [^125^I] crustacean hyperglycemic hormone (CHH) crosslinked to the specific binding sites in the membranes of the J-YO and hepatopancreas suggests a molecular weight of ~51 kDa for a MIH receptor in both tissues and a molecular weight of ~61 kDa for a CHH receptor in the hepatopancreas. The use of an *in vitro *incubation of hepatopancreas fragments suggests that MIH probably utilizes cAMP as a second messenger in this tissue, as cAMP levels increased in response to MIH. Additionally, 8-Bromo-cAMP mimicked the effects of MIH on vitellogenin (*VtG*) mRNA and heterogeneous nuclear *(hn) VtG *RNA levels. The results imply that the functions of MIH in the regulation of molt and vitellogenesis are mediated through tissue specific receptors with different kinetics and signal transduction. MIH ability to regulate vitellogenesis is associated with the appearance of MIH specific membrane binding sites in the hepatopancreas upon pubertal/final molt.

## Background

The X-organ in the eyestalks of crustaceans produces a family of crustacean hyperglycemic hormone (CHH) neuropeptides unique to arthropods. The CHH family members in crustaceans (CHH, molt-inhibiting hormone (MIH), mandibular organ-inhibiting hormone (MOIH), and gonad/vitellogenesis-inhibiting hormone (GIH/VIH)), are involved in the regulation of a variety of physiological processes [1-6]. It has been established that individual CHHs are multifunctional, having specific binding sites in multiple target tissues [7,8]. More specifically, in addition to its primary hyperglycemic action [9], CHH inhibits ecdysteroidogenesis [10]; regulates water uptake during ecdysis [6]; and inhibits methionine incorporation in ovarian fragments, *in vitro *[11]. It was recently demonstrated that in addition to its traditional molt inhibiting role, MIH is also involved in the regulation of vitellogenesis in the mature female *Callinectes sapidus *[12] and in *Metapenaeus ensis *[13]. Since specific hormonal functions are accomplished by neuropeptides and their corresponding receptors [14], efforts have been made to identify and characterize the CHH neuropeptides family of receptors by means of binding kinetics [15-18], signal transduction [15,19-27], and cloning studies [28-31]. However, to date, the mechanisms by which the multiple functionality of these neuropeptides is being executed, are not fully understood.

MIH exerts its molt inhibiting activity on the Y-organs (YO) through the suppression of ecdysteroid synthesis and secretion [32-36] via the down-regulation of protein synthesis [19,22]. It has been reported that MIH binds exclusively to a YO membrane receptor with high affinity in a specific, displaceable, and saturable manner [16,17]. Attempts to define the mechanism of MIH signaling revealed changes in YO responsiveness throughout a molt cycle [23,37,38]. While MIH titers in the hemolymph and the number of binding sites of MIH in the YO of *Carcinus maenas *remained unchanged throughout a molt cycle, the level of cGMP responding to MIH in this tissue was greater at intermolt than at premolt stages [38]. This was further supported by the finding in *Procambarus clarkii *that phosphodiesterase activity in the YO at intermolt is markedly low, resulting in an extended cyclic nucleotide life span and in turn, higher levels [23].

It has been reported that MIH and CHH act via cGMP as a primary second messenger [15,23,26-28,37-39] although the involvement of cAMP, or both cAMP and cGMP [25] was also suggested. The large increase in cGMP production in the YO in response to MIH of *C. sapidus *[23] and the stimulation of cGMP in many tissues by CHH in *C. maenas *[15] have implicated a potential involvement of nitric oxide (NO^-^), soluble guanylate cyclases [20], or membrane guanylate cyclase type receptors [28,31,40]. However, the structural characterization of a receptor for the CHH family of neuropeptides has not yet been elucidated in crustaceans nor in insects [41].

Ovarian development in crustaceans is controlled by neurohormones [42,43]. The inhibition of vitellogenesis by sinus gland factors is described in many species [3,44,45], while gonad stimulatory activity originates in the eyestalk, brain, and thoracic ganglia [13,46,47]. Overall, it appears that inhibition and stimulation are mediated by neuropeptides of the CHH family [13,44,45,47-53], with one exception that attributes the stimulatory action to a small peptide of 1000–2000 Da [54]. Further, a study in *Marsupenaeus japonicus *suggests that vitellogenic inhibition is modulated by Ca^2+^, cAMP, cGMP, and protein kinase C [24]. Much work is still required, however, to define the exact second messengers and the signal transduction pathways downstream of the neuropeptides that control vitellogenesis in crustaceans.

We have recently reported that MIH levels in the hemolymph of female *C. sapidus *are correlated with vitellogenic activity, i.e., higher at mid-vitellogenic than at previtellogenic stage. Furthermore, by the incubation of hepatopancreas fragments *in vitro*, we demonstrated that MIH acts directly in an ovarian stage dependent manner on the hepatopancreas where it stimulates mRNA, heterogeneous nuclear RNA of vitellogenin (*hnVtG *RNA = the newly transcribed yet unprocessed nuclear *VtG *mRNA) and VtG translation [12]. Consequently, we proposed that MIH has a regulatory role in vitellogenesis of the adult female, in addition to its prototypical molt inhibitory function. This fact imposed a re-examination of the accepted paradigm that MIH binds exclusively to the membranes of YO [16,17] by verifying the presence of a specific receptor for MIH in the hepatopancreas of the female *C. sapidus*.

In the current study, we employed a classical radioligand binding assay to 1) locate and characterize specific binding sites of MIH in hepatopancreatic membranes of vitellogenic females, and 2) determine the molecular weights of MIH receptors in the membranes of hepatopancreas and the YO. In addition, we also examined the second messenger of MIH in hepatopancreas using an *in vitro *incubation assay and radioimmunoassays (RIA). The YO responded to MIH by elevating cGMP levels, while the hepatopancreas responded by increasing cAMP production. Altogether, our results indicate that MIH acts on the YO and the hepatopancreas via a tissue specific receptor.

## Results

### MIH binding to the YO membranes

**Figure 1:**
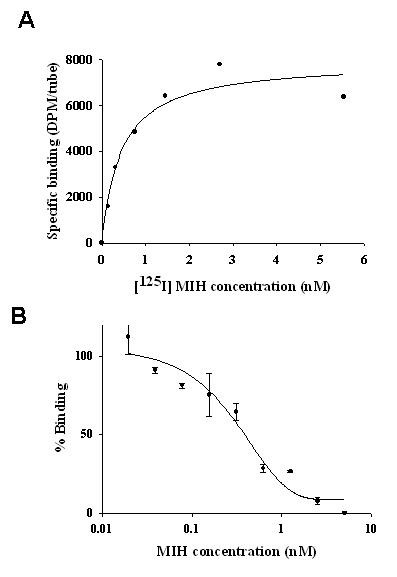
**Saturation curve (A) and displacement curve (B) of [^125^I] MIH to the membranes of YO of juvenile *C. sapdius***. Bound [^125^I] MIH was displaced with unlabelled rMIH. The data are presented as mean ± SEM of the triplicates.

To determine the maximal number of binding sites of MIH in the membranes of the YO, 50 μg of membrane proteins were incubated with [^125^I] MIH from 0.15 to 12.3 nM. The non-specific binding was determined using recombinant MIH (rMIH) due to the limited amount of native MIH (nMIH). The rMIH was as potent as nMIH in cGMP production in the YO *in vitro *(unpublished results) and produced the same EC_50 _value in a specific RIA using antibody raised against rMIH [12]. As shown in Fig. 1A, the binding sites were saturable with a K_D _value of 4.19 × 10^-10 ^M/mg protein and a B_MAX _value of 4.8 × 10^-9 ^M/mg protein. Labeled and bound MIH was displaced with cold rMIH [12] at concentrations ranging from 24.4 pM to 10 nM (Fig. 1B) with an EC_50 _value of 0.67 nM. A displacement study was carried out on YO membranes using cold nMIH, resulting in a similar EC_50 _value of 0.66 nM. The non-specific binding in these experiments was ~20–30% of the total binding.

### MIH binding to the hepatopancreas membranes

**Figure 2:**
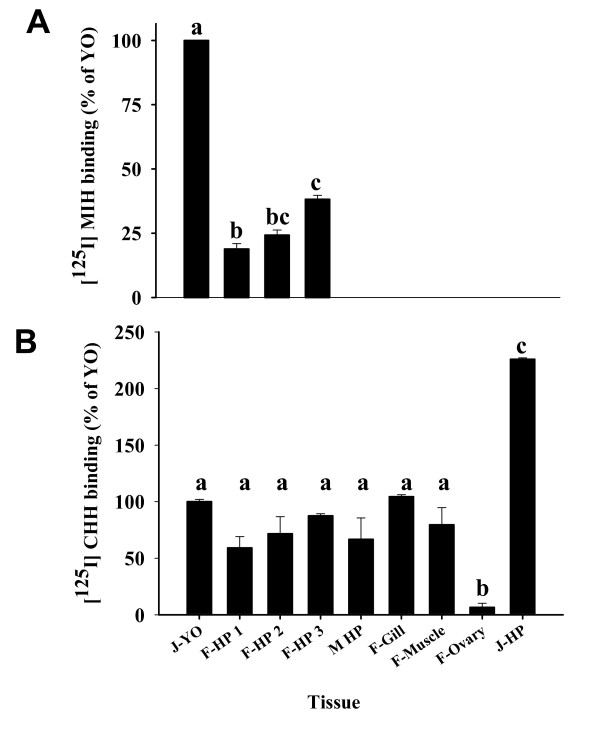
**MIH specifically binds to membranes of mature female hepatopancreas with higher binding at mid-vitellogenic stage than pre-vitellogenic**. A) Specific binding of [^125^I] MIH to various tissue membranes of vitellogenic females and YO and hepatopancreas of juveniles. B) Specific binding of [^125^I] CHH to the same membranes. All membranes were prepared from five animals, except for J- YO membranes that were prepared from 700 intermolt animals. F, Females; M, Males; J, juveniles; HP, hepatopancreas; 1, 2, and 3 refer to the ovarian stages; F-ovary- ovarian membrane of females at ovarian stage 3. Results are presented as mean ± SEM of the triplicates as % of the J-YO. The alphabetical letters show the significant differences at P < 0.05.

Membranes (100 μg) of hepatopancreas at ovarian stages 1 to 3 and gills, abdominal muscle, YO and ovaries that were pooled from five females at each ovarian stage were incubated with 0.4 pmol [^125^I] MIH (263,000 DPM) with or without an excess of 20 pmol unlabelled rMIH. Hepatopancreas membranes prepared from five adult males and from juveniles were also tested, with juvenile YO (J-YO) membranes serving as a reference control. Specific binding of MIH was found only in mature female hepatopancreas among the tissues tested. MIH binding at ovarian stage 3 was two times higher than in stage 1 and comprised ~40% of the J-YO control (Fig. 2A). MIH binding to the YO of mature females at stage 3 was equal to that of juveniles (not shown in the figure). The same membranes were tested for [^125^I] CHH binding by incubating 100 μg with 0.12 pmol [^125^I] CHH (~182,000 DPM) and unlabeled native CHH at 20 pmol for non-specific binding. No significant difference in CHH binding to the hepatopancreas was observed between the different ovarian stages, however, CHH binding to juvenile hepatopancreas was two times higher than to J-YO and the other tissues. The ovaries exhibited a binding 20 times lower than J-YO (Fig. 2B).

**Figure 3:**
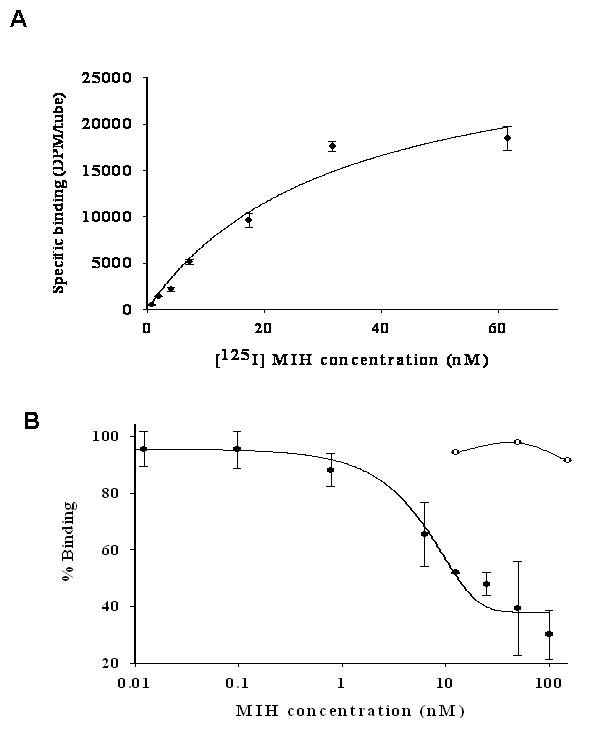
**Kinetic binding studies of [^125^I] MIH to the hepatopancreas membranes of female *C. sapidus *at ovarian stage 3 demonstrate receptor/ligand typical and specific binding**. Five membrane preparations were pooled and tested. A) Saturation curve; B) Displacement curve. [^125^I] MIH binding sites were displaced with unlabelled rMIH (closed circles) or unlabelled CHH (open circles). The data are presented as mean ± SEM of the triplicates.

A saturation curve was obtained by incubating 0.75 to 60 nM [^125^I] MIH with 100 μg hepatopancreas membranes of ovarian stage 3 females (Fig. 3A). The calculated K_D _and B_MAX _values were 3.22 × 10^-8 ^M/mg protein and 9.24 × 10^-9 ^M/mg protein, respectively. Displacement studies were conducted using 0.04 nM – 100 nM unlabeled rMIH as well as 12.5 – 100 nM native CHH. rMIH competed with [^125^I] MIH on the binding sites with an EC_50 _value of 17.35 nM, whereas CHH showed no displacement of [^125^I] MIH (Fig. 3B). The non-specific binding in these studies was ~40% for MIH and ~30% for CHH.

**Figure 4:**
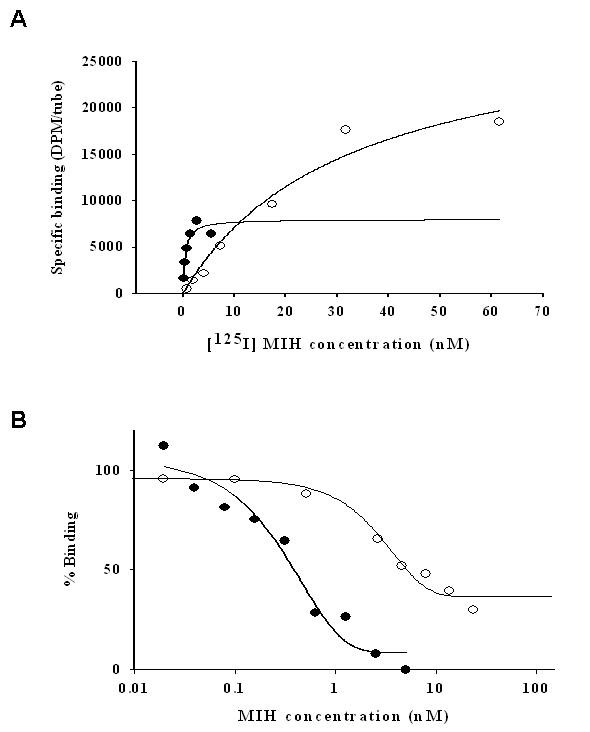
**MIH binding to female hepatopancreas membranes is characterized by a lower affinity and higher density compared to juvenile YO**. Overlay of the curves of saturation (A) and displacement (B) of [^125^I] MIH binding to YO and female hepatopancreas membranes to emphasize the differences in values of K_D _and B_MAX _between these tissues. YO, closed circles; hepatopancreas, open circles.

The plots obtained for MIH binding to the membranes of the J-YO and hepatopancreas were overlaid in order to demonstrate the difference in the affinities and the number of maximal binding sites (Fig. 4A and 4B). Calculated K_D _values were ~77 times greater in the J-YO than female hepatopancreas: 4.19 × 10^-10 ^and 3.22 × 10^-8 ^M/mg protein, respectively. Values of B_MAX _were 9.24 × 10^-9 ^M/mg protein for hepatopancreas and 4.80 × 10^-9 ^M/mg protein for J-YO.

### MIH and CHH effects on cAMP and cGMP levels in the hepatopancreas of vitellogenic females, *in vitro*

**Figure 5:**
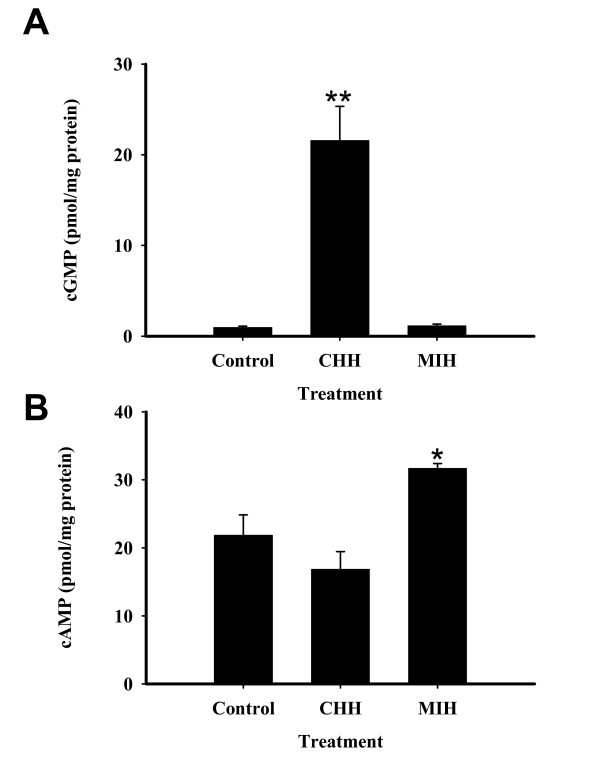
**CHH induces cGMP and MIH induces cAMP production in female hepatopancreas fragments**. *In vitro*: A) cGMP; B) cAMP. Hepatopancreas fragments were incubated with 20 nM CHH or 2 nM MIH. The results are presented as mean ± SEM (N = 4). *, P ≤ 0.05; **, P ≤ 0.01.

The effects of MIH and CHH on levels of cAMP and cGMP production were tested *in vitro *in hepatopancreas fragments of vitellogenic females at ovarian early stage 2 (E2). As presented in Figs. 5A and 5B, CHH (20 nM) resulted in a 16 times increase in the levels of cGMP production (from 2 to 32 pmol/mg protein), while MIH at 2 nM had no effect. Cyclic AMP levels did not change with CHH, but increased by 50% in response to 2 nM MIH (from 22 to 33 pmol/mg protein, N = 4), compared to the control which received 1 mM isobutylmethylxanthine (IBMX) alone (Fig. 5B).

### The effects of cyclic nucleotide analogs on *VtG *transcriptions: *hnVtG *RNA and *VtG *mRNA

**Figure 6:**
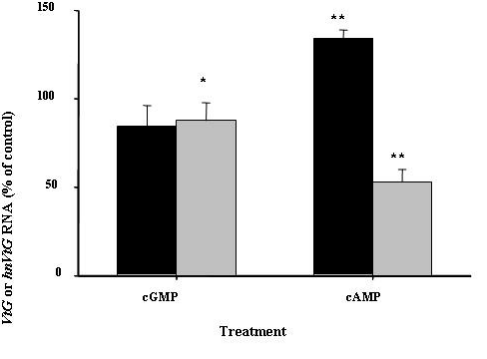
**cGMP analog mimics the effects of CHH and cAMP analog mimics the effect of MIH on *VtG *mRNA and *hnVtG *RNA in the hepatopancreas fragments of stage E2 females, respectively**. Hepatopancreas fragments were incubated with 10 μM 8-Bromo- cGMP or 8-Bromo- cAMP followed by QPCR analysis to detect changes in *hnVtG *(Black) and *VtG *mRNA (Grey). The results are presented as mean ± SEM of % of control (N = 4). *, P ≤ 0.05; **, P ≤ 0.01.

The incubation of hepatopancreas fragments at E2 with the membrane permeable 8-Bromo-cAMP resulted in a 36% increase in the level of *hnVtG *RNA, while 8-Bromo-cGMP had no effect (Fig. 6). *VtG *mRNA in the hepatopancreas at E2 decreased to 50% compared to the control with 8-Bromo-cAMP, whereas it remained constant with 8-Bromo-cGMP (Fig. 6).

### Molecular weights of putative MIH receptors in the membranes of the YO and hepatopancreas of vitellogenic female

**Figure 7:**
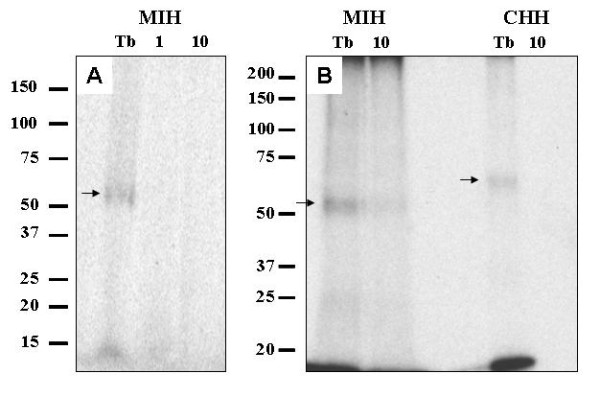
**Binding sites of [^125^I] MIH in the membranes of female's hepatopancreas and juvenile YO have a similar molecular mass of ~51 kDa, which is different from that of [^125^I] CHH in the hepatopancreas**. A) J-YO (100 μg); B) female hepatopancreas at ovarian stage 3 (200 μg). Membranes were incubated without (total binding, Tb) or with unlabeled MIH or CHH in an excess of 1 or 10 pmol, specified at the top of the lane. YO membranes were resolved on a 4–15% SDS-PAGE and hepatopancreas membranes on a 10% SDS-PAGE. Labeled ligand and receptor complexes are indicated with arrows.

YO and hepatopancreas membranes that were preincubated with [^125^I] MIH or hepatopancreas with [^125^I] CHH and crosslinked with disuccinimidyl suberate (DSS), were separated on SDS-PAGE and the signal was detected using a phosphorimager. Two bands were observed for each membrane: a lower one at the expected size of MIH (9070.9 Da) or CHH (8478.1 Da) [55]; and, a second higher band at an estimated size of ~60 kDa for MIH (Figs. 7A and 7B). Binding of [^125^I] CHH to hepatopancreas membranes revealed a signal at a molecular weight of ~70 kDa (Fig. 7B). Assuming a binding ratio of 1 to 1 (ligand: receptor), the estimated size for the MIH receptor in both the YO and the hepatopancreas is ~51 kDa and ~61 kDa for the CHH receptor in the hepatopancreas.

## Discussion

Based on our recent finding that MIH acts as a vitellogenesis stimulant in addition to its molt inhibitory function [12], we demonstrated in the current study the presence of specific binding sites for MIH in the J-YO membranes as well as novel binding sites in hepatopancreatic membranes of vitellogenic female *C. sapidus*. Kinetic studies revealed a specific, saturable, and non-cooperative binding indicative of a receptor-ligand interaction in both tissues, with differences in the affinity and density of the receptors between the two tissues. In addition, we have shown by crosslinking [^125^I] MIH to its receptor in the YO and hepatopancreas that the receptors are proteins with an estimated molecular weight of ~51 kDa.

The radioligand receptor assays revealed that the presence of MIH specific binding sites in the hepatopancreas is found only in adult females and is vitellogenic stage- specific. Bound MIH in the hepatopancreas was specifically displaced by recombinant MIH (rMIH), but not by CHH (Fig. 3B). Moreover, MIH binding capacity in the hepatopancreas increased as ovarian stage advanced from 1 to 3, reaching ~40% of that of the J-YO binding, reflecting B_MAX _values (4.8 × 10^-9 ^and 9.24 × 10^-9 ^M/mg protein of J-YO and hepatopancreas, respectively). The ovarian stage dependent increase of MIH binding in the hepatopancreas may be responsible for the ovarian stage dependent MIH effect on vitellogenesis[12]. In contrast, CHH binding was ubiquitous in the tissues tested except for ovary, as has been described in *C. maenas *[15]. Moreover, CHH binding in the hepatopancreas did not differ significantly between ovarian stages and sex (Fig. 2B). The B_MAX _value for CHH binding in the hepatopancreas of *C. sapidus *was 3.67 × 10^-10 ^M/mg protein [18], similar to the value of 3.28 × 10^-10 ^M/mg protein obtained for hepatopancreas of *C. maenas *[15]. This suggests that at ovarian stage 3, the number of MIH receptors in the hepatopancreas is the highest ever reported in the binding studies of the family of CHH neuropeptides, being 30 times higher than that of the CHH receptor. This high density of MIH receptors in hepatopancreas may cause higher non-specific binding than in the J-YO, as shown in Fig, 4B and Figs 7A and 7B. When considering the difference in size of the tissues between the J-YO (~20 mg) and the hepatopancreas (~7 g) in adult females, the total number of MIH binding sites can easily be ~700 times higher in the hepatopancreas than in the YO.

As shown in Fig. 4, K_D _values of MIH binding sites in the J-YO and female hepatopancreas membranes were significantly different: 4.19 × 10^-10 ^and 3.22 × 10^-8 ^M/mg protein, respectively. These values indicate that the affinity of the MIH receptor in the hepatopancreas is 77 times lower than that of the J-YO. Similar to what has been reported for *C. maenas*, CHH binding sites in the YO and hepatopancreas membranes had calculated K_D _values of 1.82 × 10^-9 ^and 6 × 10^-10 ^M/mg protein, respectively [17]. These affinities were comparable to those obtained for MIH binding sites in the J-YO (4.19 × 10^-10 ^M/mg protein), all in the 0.1 nM/mg protein range. Thus, it seems that MIH binding sites in the hepatopancreas have the lowest affinity to its ligand among those of CHH neuropeptides characterized thus far. The low affinity of the hepatopancreas receptor may require high circulating titers of MIH, with concentrations ranging from 0.01–20 nM for binding (Fig. 3A). The level of MIH at ovarian stage 3 was ~0.02 nM [12], which was slightly higher than that of intermolt (~0.015 nM). Taken together, MIH receptor in the hepatopancreas is featured by low affinity-high binding capacity to its ligand (Fig. 4). The presence of different receptors for a ligand with different binding kinetics is well known for steroid receptors such as glucocorticoid receptor [56], peptides like gonadotropin-releasing hormone receptors [57], and polypeptide receptors such as prolactin receptor [58].

To determine whether the MIH signals through cGMP as a second messenger in the hepatopancreas as it does in the YO [38], we measured *in vitro *production of cyclic nucleotides in hepatopancreas fragments in the presence of 2 nM MIH or 20 nM CHH, which are effective doses in vitellogenesis [12]. As expected, intracellular cGMP levels in the hepatopancreas increased by 16 fold in response to CHH, but did not change with MIH treatment (Fig. 5A). On the other hand, cAMP levels significantly increased by 50% with MIH, but did not change with CHH treatment (Fig. 5B). The difference in the magnitude of response in the increase of cGMP (by CHH) compared to cAMP (by MIH) may be due to different basal levels: cAMP, 20 pmol/mg protein; cGMP, < 1 pmol/mg protein. Hepatopancreas fragments incubated with 8- Bromo-cAMP augmented *hnVtG *RNA levels, while 8-Bromo-cGMP had no effect (Fig. 6). These findings are congruent with our previous results of no effect of CHH and the stimulatory effect of MIH on *hnVtG *transcription at stage E2 [12]. Moreover, the membrane permeable 8-Bromo-cAMP and 8-Bromo-cGMP mimicked the effects of MIH and CHH, respectively, on *VtG *mRNA. *VtG *mRNA significantly decreased by 60% with MIH and 20% with CHH in hepatopancreas fragments of ovarian stage E2 females[12].

The results obtained from the crosslinking studies showed that the complexes of MIH and its receptor in both the J-YO and the hepatopancreas have an estimated size of ~60 kDa, resulting in a MW of ~51 kDa for the receptor, which is different from that of the CHH receptor in the hepatopancreas (MW ~61 kDa). Together with the second messenger data, this suggests that there is a tissue specific type of membrane receptor for MIH, since only one band appeared in the autoradiogram in addition to the unbound neuropeptides (Figs. 7A and 7B). A similar study estimated the size of the MIH receptor in the YO of the kuruma prawn as ~70 kDa [16]. Such a difference may lie in the difference in species.

The increase in intracellular cAMP in the hepatopancreas caused by MIH opposes the possibility that the receptor is a guanylate cyclase, and instead favors the option of a G protein – coupled receptor (GPCR) that acts through a GTP binding protein to activate a nucleotide cyclase. This agrees with the calculated MW of the hepatopancreas putative MIH receptor of ~51 kDa, which is different from the typical membrane guanylate cyclase having a MW of 120–140 kDa [59]. In addition, the similarity in the MWs of MIH binding sites on the YO and hepatopancreas indicates that both MIH receptors may be GPCRs, however, each form may activate a different signaling pathway. A possible scenario is that the hepatopancreas MIH receptor activates the α_s _subunit of the G protein-adenylyl cyclase, while the YO isoform activates soluble guanylyl cyclase through Ca^2+^and NO^- ^initiated by an increase in cAMP. Support for the latter option is found in a study reporting that incubation of the YO of *C. maenas *and *P. clarkii *at premolt stage with MIH resulted in a sustained 60 times increase in cGMP levels, and also a short, transient two fold increase in cAMP [23,25]. In addition, it was suggested that nitric oxide synthase and NO^- ^are possibly involved in the MIH signaling in the YO of the land crab, *Gecarcinus lateralis *[20,60]. In this regard, the involvement of trimeric G proteins in inhibition of protein synthesis in the YO of *C. sapidus*, was proposed [29]. Overall, the two suggested signaling pathways may involve a complex network of interactions and will require more rigorous investigations.

It was speculated that an extensive gene duplication event may have occurred to produce multiple CHH isoforms which may show tissue specific expression [61]. Similarly, receptors including the GPCR family members are believed to propagate by gene duplication from a common ancestor [62]. This includes odorant receptors [63], hormone receptors like growth hormone [64], gonadotrophins [65], gonadotrophin releasing hormones [66] and many more. Although the sequences of the MIH receptors in the current study are still unknown, these two receptors may possibly be products of gene duplication and share a high degree of structure similarity.

It is generally recognized that individual hormones can elicit several diverse responses in different organs and tissues, as well as in individual cells [14]. Several mechanisms may be involved in mediating the different signals such as 1) pulses and micropulses (frequency of changes of hormone circulation or endogenous concentrations); 2) changes in the activities of hormone-converting enzymes; 3) selective activation of each receptor type, subtype, and isoform; 4) changes in receptors, which may activate and suppress different signaling pathways (e.g. phosphorylation, dimerization); and 5) activation and suppression of different nuclear transcription processes [14]. The MIH receptor in the mature female's hepatopancreas is clearly different from the one present in the YO in terms of its time of appearance/functionality, its binding kinetics, and signal transduction. Thus, it fits some of the mechanisms mentioned above and provides a possible model for multi-functionality of the CHH neuropeptide family, which is based upon a diverse spatial and temporal expression, binding kinetics and signal transduction pathway. Based on this finding, we propose that some pleiotropicity of the CHH neuropeptide family might be mediated by multiple receptor forms: each form executes a different function.

## Conclusion

In this study, we demonstrated the presence of novel specific MIH binding sites in the hepatopancreas of the mature female *C. sapidus *that are involved in MIH regulation of vitellogenesis. MIH action in the hepatopancreas is probably mediated by cAMP, unlike the YO counterpart that utilizes cGMP as a second messenger. Our result suggests that in the female *C. sapidus*, the antagonism between molting and reproduction is mediated by MIH that acquires a vitellogenesis stimulating function in addition to its traditional molt-inhibitory action. This new role is obtained through the expression of abundant MIH specific receptors in the hepatopancreas of the adult females. To our knowledge, this is the first description of an endocrine regulation mechanism of the antagonism between molting and reproduction in a crustacean species. As we proposed in Zmora et al. [12], this may occur in other crustaceans, particularly in those who share a similar life cycle with the female *C. sapidus *(i.e., a terminal molt upon puberty). It will be interesting to further examine how the two MIH receptors structurally differ and determine the specific timing and cues for the hepatopancreas MIH receptor appearance or activation.

## Methods

### Animals

Juvenile blue crabs at intermolt stage (carapace width 5–7.5 cm) were collected by using a seine or a trot line from the eastern shore of the Chesapeake Bay [67]. Mature females obtained from a local waterman were transferred in aerated water and acclimated for 2 to 3 days without feeding in a 4.5 cubic meter re-circulating tank at ambient conditions. Tissue collection was carried out as described [68]. The tissues were dissected, rinsed in ice-cold crustacean saline and stored at -80°C until further processing.

### Purification and quantification of native CHH, native MIH, and recombinant MIH

Neuropeptides of the sinus glands (SG) and rMIH were purified using RP-HPLC as described [17]. Amino acid analyses were carried out for the quantification of the purified native MIH, native CHH and rMIH using *o*-phthalaldehyde pre-column derivatization method as outlined [17].

### Radioligand binding assays

#### Preparation of membranes

Membranes were prepared from ~700 YO's collected from juvenile *C. sapidus *(J-YO, 5–7.5 cm carapace width) at intermolt stage (C4) by following the method as described [17]. Hepatopancreas membranes were prepared from individual females or males, using ~1 g tissue. Each hepatopancreas or 700 pooled YO were homogenized in 20 ml ice-cold homogenization buffer (140 mM NaCl, 300 mM sucrose, 10 mM HEPES, and 10 mM benzamidine (Sigma), pH 7.4) using an Ultra Torax homogenizer. After an initial centrifugation at 1000 g for 5 min at 4°C, the supernatant was pelleted by centrifugation at 30,000 g for 30 min at 4°C. The pellet was washed in washing buffer (140 mM NaCl and 10 mM HEPES, pH 7.4 without BSA) by repeating the previous centrifugation step for 15 min and resuspending in the same buffer. Protein concentration was determined using a D_C _protein quantification kit (BioRad) and the membranes were aliquoted and stored at -80°C until further use.

### MIH and CHH binding studies

#### Binding assays

Native [^125^I] MIH and native [^125^I] CHH were prepared using the chloramine-T labeling method as described [17] or using 1,3,4,6-tetrachloro-3alpha, 6alpha-diphenylglucoluril (iodogen) coated tubes (Pierce) according to the manufacturer's instruction. [^125^I] MIH or [^125^I] CHH was separated from free [^125^I] on a PD 10 column (GE Healthcare) as described [17]. Specific activities were approximately 300–500 Ci/mmol.

The MIH binding procedure was followed as stated [69]. Briefly, membranes were incubated in binding assay buffer (washing buffer containing 1% BSA) with [^125^I] MIH or [^125^I] CHH for 1 h at room temperature. For displacement and non-specific binding, unlabeled native CHH or recombinant MIH (rMIH) produced in S2 *Drosophila *cells (Zmora et al. companion paper 1) was added to the reaction. The membranes were then washed in binding assay buffer and pelleted by centrifugation at 14,000 rpm for 5 min. The pellets were counted in a gamma-counter (HP counter). Each assay was repeated three or four times in triplicates.

### The effects of MIH and CHH on the levels of cAMP and cGMP, *in vitro*

The hepatopancreas of vitellogenic females at stage E2 was excised and washed in 10 volumes of ice-cold Medium 199 (osmolarity 960 mmol/kg, 0.1 mg/ml BSA and 1× protease inhibitors cocktail for tissue culture (Sigma), pH 7.4) for 3 h on ice, with three media changes as described [68]. Quantification of cAMP or cGMP in tissues using RIAs followed the procedure as described [70]. In brief, fragments of 10 mg each were incubated for 1 h at room temperature in the presence of 2 nM MIH or 20 nM CHH in 400 μl Medium199 medium containing 1 mM IBMX, or only IBMX for the control. The tissues were then disrupted by sonication (Branson) and centrifuged at 14,000 rpm for 10 min at 4°C. Supernatants (100 μl) were transferred to tubes containing 900 μl of 0.1 M acetate buffer (pH 4.75) and immediately acetylated by the addition of 20 μl triethylamine (Sigma) and 10 μl acetic anhydride (Sigma). Fifty or one hundred μl of acetylated samples were subjected to cAMP and cGMP RIAs. The standards for cAMP and cGMP in the range of 1–1000 fmol/tube were treated the same as the samples. The final dilution of antibodies was 1: 21,000 for cGMP and 1:4000 for cAMP.

2'-O-methyl ester cAMP or cGMP (0.3 nmol) (Sigma) were iodinated with Na [^125^I] (Amersham) using the chloramine T method as described [70]. The iodinated material was separated from free [^125^I] on a C_18 _Sep-Pak cartridge (Waters) by elution with 40% isopropanol [71]. The calculated specific activities were approximately 500–600 Ci/mmol.

### The effects of membrane permeable analogues of cAMP and cGMP on vitellogenin gene expression, *in vitro*

Hepatopancreas tissue was washed as described above and fragments of 10 mg each were incubated in 400 μl Medium199 medium containing 10 μM 8-Bromo-cAMP or 8-Bromo-cGMP (Alexis) for 1 or 6 h. RNA was extracted and the levels of *hnVtG *RNA and *VtG *mRNA were determined using quantitative PCR analysis (QPCR) analysis as described in Zmora et al. (companion paper 1).

### Crosslinking [^125^I] MIH or [^125^I] CHH to their binding sites and visualization

Juvenile YO membranes (100 μg) were incubated with 0.4 pmol [^125^I] MIH (~263,000 DPM) for total binding or with additional 1 or 10 pmol/100 μl unlabeled rMIH for nonspecific binding. The membranes were then washed twice with 1 ml ice-cold washing buffer by centrifugation at 14,000 rpm for 10 min. The pellets were resuspended in 100 μl buffer containing DSS (Pierce) at a final concentration of 1 mM for 30 min at room temperature. The reaction was then quenched by the addition of 15 μl of 1 M Tris buffer (pH 7.4) for 15 min at room temperature and centrifuged at 14,000 rpm for 5 min. The pelleted membranes were resuspended in 1× SDS sample loading buffer (Bio-Rad) and denatured for 5 min at 100°C. Proteins were separated on a 4–15% SDS-PAGE and briefly stained with Bio-Safe coomassie (Bio-Rad) for visualization. The gel was then dried and exposed to a Phosphor-imager screen for 2 h at room temperature and analyzed using a Typhoon 9410 variable mode imager (Molecular Dynamics).

As for hepatopancreas membranes, the conditions described above were applied except that 200 μg of the hepatopancreas membranes were incubated with 1 pmol [^125^I] MIH (~657,000 DPM) with or without 10 pmol cold MIH or 0.12 pmol [^125^I] CHH (182,500 DPM) and 10 pmol cold CHH for non-specific binding. After crosslinking with 5 mM DSS, membranes were lysed by adding 4% Triton X-100 for 10 min in binding buffer, followed by dilution to 1% Triton X-100. The sample was then immunoprecipitated with 4 μl MIH antiserum for 1 h at 4°C, followed by the addition of protein-A magnetic beads (New England Biolabs) to the mixture and incubated for an additional hour. Bound proteins were separated via a magnet apparatus and eluted with 3× SDS sample loading buffer. After heating at 70°C for 3 min, the samples were run on a 10% SDS PAGE. The gel was dried and analyzed as described above.

### Statistical analysis

The results obtained from the radioligand binding studies are presented for each experiment as the mean ± SEM of the experimental replicates. The data obtained from QPCR analysis and RIA are presented as mean ± SEM for the separate experiments. The results were subjected to GraphPad Instat 3 program analysis (Graphpad) and were examined using one-way ANOVA followed by the Tukey-Kramer multiple comparison test. In all cases, statistical difference was accepted at P ≤ 0.05.

## Abbreviations

MIH: molt-inhibiting hormone; CHH: crustacean hyperglycemic hormone; QPCR: quantitative PCR; *VtG*: vitellogenin mRNA; VtG: vitellogenin protein; cAMP: 3'-5'-cyclic adenosine monophosphate; cGMP; 3'-5'-cyclic guanosine monophosphate.

## Competing interests

The authors declare that they have no competing interests.

## Authors' contributions

NZ carried out the concept, experimental design, and acquisition, analyses, and interpretation of data, and drafted and revised the manuscript including tables and figures. CJS was involved in the acquisition of funding, contributed to concept, experimental design, analyses, and interpretation of data, and revised the manuscript. AS participated in the discussions and revision of the manuscript. YZ was involved in the acquisition of funding and contributed in discussions.

All authors read and approved the final manuscript.
